# Statistical Inference of the Half-Logistic Inverse Rayleigh Distribution

**DOI:** 10.3390/e22040449

**Published:** 2020-04-15

**Authors:** Abdullah M. Almarashi, Majdah M. Badr, Mohammed Elgarhy, Farrukh Jamal, Christophe Chesneau

**Affiliations:** 1 Statistics Department, Faculty of Science, King AbdulAziz University, Jeddah 21577, Saudi Arabia; aalmarashi@kau.edu.sa; 2 Statistics Department, Faculty of Science for Girls, University of Jeddah, Jeddah 21577, Saudi Arabia; mmbadr@uj.edu.sa; 3 Valley High Institute for Management Finance and Information Systems, Obour, Qaliubia 11828, Egypt; m_elgarhy85@sva.edu.eg; 4 Department of Statistics, Government Postgraduate College Der Nawab Bahawalpur, Punjab 63351, Pakistan; drfarrukh1982@gmail.com; 5 Department of Mathematics, LMNO, Campus II, Science 3, Université de Caen, 14032 Caen, France

**Keywords:** inverse Rayleigh distribution, half-logistic transformation, moments, entropy, statistical inference, real data analysis, 60E05, 62E15, 62F10

## Abstract

The inverse Rayleigh distribution finds applications in many lifetime studies, but has not enough overall flexibility to model lifetime phenomena where moderately right-skewed or near symmetrical data are observed. This paper proposes a solution by introducing a new two-parameter extension of this distribution through the use of the half-logistic transformation. The first contribution is theoretical: we provide a comprehensive account of its mathematical properties, specifically stochastic ordering results, a general linear representation for the exponentiated probability density function, raw/inverted moments, incomplete moments, skewness, kurtosis, and entropy measures. Evidences show that the related model can accommodate the treatment of lifetime data with different right-skewed features, so far beyond the possibility of the former inverse Rayleigh model. We illustrate this aspect by exploring the statistical inference of the new model. Five classical different methods for the estimation of the model parameters are employed, with a simulation study comparing the numerical behavior of the different estimates. The estimation of entropy measures is also discussed numerically. Finally, two practical data sets are used as application to attest of the usefulness of the new model, with favorable goodness-of-fit results in comparison to three recent extended inverse Rayleigh models.

## 1. Introduction

In the seventies, [1] introduced what will be reveal as an important distribution for lifetime and reliability studies, known as inverse Rayleigh (IR) distribution. Specially, it provides an appropriate statistical model when dealing with unimodal highly right-skewed data. As mathematical basis, the corresponding cumulative distribution function (cdf) and probability density function (pdf) are given by

(1) $$\begin{array}{c} G\left(x;\alpha \right)=e^{-{\left(\frac{\alpha }{x}\right)}^{2}},\;g\left(x;\alpha \right)=\frac{2\alpha^{2}}{x^{3}}e^{-{\left(\frac{\alpha }{x}\right)}^{2}},\;x,\alpha >0, \end{array}$$

respectively, where α is a scale parameter. As notable features, the IR distribution has tractable and simple probability functions, is unimodal and right-skewed, and possesses a hazard rate function with a singular curvature: it increases at a certain value, then decreases until attain a kind of stabilization. The pioneer studies are [2] which presents some properties of the maximum likelihood estimator of α, and [3] which provides closed-form expressions for the (standard) mean, harmonic mean, geometric mean, mode and median of the IR distribution. Also, among the amount of works investigating the statistical aspects of the IR distribution, the reader can be referred to [4,5,6,7,8,9,10,11,12].

In the recent years, several extensions of the IR distribution were developed, using different mathematical techniques, often at the basis of general families of distributions. Among them, there are the beta IR (BIR) distribution by [13], transmuted IR (TIR) distribution by [14], modified IR (MIR) distribution by [15], transmuted modified IR (TMIR) distribution by [16], transmuted exponentiated IR (TEIR) distribution by [17], Kumaraswamy exponentiated IR (KEIR) distribution by [18], weighted IR (WIR) distribution by [19], odd Fréchet IR (OFIR) distribution by [20], type II Topp-Leone IR (TIITLIR) distribution by [21], type II Topp-Leone generalized IR (TIITLGIR) distribution by [22] and exponentiated IR (EIR) distribution by [23].

However, to the best of our knowledge, the use of the half-logistic transformation to extend the IR distribution remains unexplored, despite recent success in this regard. This half-logistic transformation was pioneered by [24] in the context of the half logistic generated (HL-G) family of continuous distributions. As main functions, the cdf and pdf of the HL-G family are, respectively, given by

(2) $$\begin{array}{c} F\left(x;\lambda ,\xi \right)=\frac{1-{\left[1-G(x;\xi )\right]}^{\lambda }}{1+{\left[1-G(x;\xi )\right]}^{\lambda }} \end{array}$$

and

(3) $$\begin{array}{c} f\left(x;\lambda ,\xi \right)=\frac{2\lambda g\left(x;\xi \right){\left[1-G(x;\xi )\right]}^{\lambda -1}}{{\left\{1+{\left[1-G(x;\xi )\right]}^{\lambda }\right\}}^{2}},\;x,\lambda >0, \end{array}$$

where λ is a shape parameter, G(x;ξ) is a cdf of a baseline/parent continuous distribution, with corresponding pdf g(x;ξ), and ξ represents the baseline parameters (under a vector form, say $\xi =(\xi_{1},\xi_{2}\ldots )$). Thus, many studies used the HL-G family to introduce new flexible continuous distributions with modelling perspectives, such as [25] with the HL Lomax (HLL) distribution, [26] in which different methods of estimation for the HLL distribution are proposed, ref. [27] with the HL power Lindley (HLPL) distribution, [28] with the HL generalized Weibull (HLGW) distribution, [29] with the HL Burr X (HLBX) distribution and [30] which studied different estimation methods for the HL Topp-Leone (HLTP) distribution.

That is, attracted by the success of the above extensions, we investigate the half-logistic IR (HLIR) distribution, constituting a new lifetime distribution with two parameters, and a new extension of the IR distribution as well. As expressed later, the cdf and pdf of the HLIR distribution are obtained by inserting (1) into (2) and (3), respectively. In view of these functions, the HLIR can also be viewed as a special case of the HL Fréchet (HLF) distribution by [24], i.e., with parameter β=2, case that not received a particular attention. The aim of this paper is to provide a solid and complete study on the HLIR distribution, with an emphasis on the statistical inference of the related model. The essential mathematical properties are provided, showing the overall flexibility of the HLIR distribution via various measures (central, dispersion, as/symmetrical, entropy…). Then, five different methods of estimation are developed for the HLIR model parameters, specifically the maximum likelihood, least square, weighted least square, percentile and Cramer-von Mises methods. The estimation of the Rényi entropy and *q*-entropy is also discussed by using the plugging and ML methods. Then, we show that the fits provided by the HLIR model can accommodate data with various features, and can demonstrate better goodness-of-fits than the three following extended IR two-parameter models: the TIITLIR model (by [21]), TIR model (by [14]) and OFIR model (by [20]), and than the former one-parameter IR model as well. Two practical data sets are analyzed in this regard.

The following sections composed the paper. Section 2 is devoted to the main probability functions of the HLIR distribution. Section 3 introduces some mathematical properties of the HLIR distribution including stochastic ordering results, a general linear representation for the exponentiated probability density function, raw/inverted moments, incomplete moments, skewness and kurtosis features, and some entropy measures. Section 4 discusses the estimation of the model parameters and entropy. In Section 5, we reveal the potential of the HLIR model compared with some other models in a concrete statistical setting. The paper encloses with some concluding remarks in Section 6.

## 2. The HLIR Distribution

This section introduces the main functions on the HLIR distribution, along with some analytical properties.

### 2.1. Probability Functions

As described in the introduction, the cdf and pdf of the HLIR distribution with the vector of parameters φ=(α,λ) is obtained by inserting Equation (1) into Equation (2) and Equation (3), i.e.,

(4) $$\begin{array}{c} F\left(x;\varphi \right)=\frac{1-{\left[1-e^{-{\left(\frac{\alpha }{x}\right)}^{2}}\right]}^{\lambda }}{1+{\left[1-e^{-{\left(\frac{\alpha }{x}\right)}^{2}}\right]}^{\lambda }} \end{array}$$

and

(5) $$\begin{array}{c} f\left(x;\varphi \right)=\frac{4\lambda \alpha^{2}e^{-{\left(\frac{\alpha }{x}\right)}^{2}}{\left[1-e^{-{\left(\frac{\alpha }{x}\right)}^{2}}\right]}^{\lambda -1}}{x^{3}{\left\{1+{\left[1-e^{-{\left(\frac{\alpha }{x}\right)}^{2}}\right]}^{\lambda }\right\}}^{2}},\;x,\lambda ,\alpha >0, \end{array}$$

respectively, where α is a scale parameter and λ is a shape parameter.

Important reliability functions of the HLIR distribution are presented below. The survival function (sf), hazard rate function (hrf), reversed hazard rate function (rhrf) and cumulative hazard rate function (chrf) of the HLIR distribution are, respectively, given by

$$\begin{array}{c} S\left(x;\varphi \right)=\frac{2{\left[1-e^{-{\left(\frac{\alpha }{x}\right)}^{2}}\right]}^{\lambda }}{1+{\left[1-e^{-{\left(\frac{\alpha }{x}\right)}^{2}}\right]}^{\lambda }}, \end{array}$$

(6) $$\begin{array}{c} h\left(x;\varphi \right)=\frac{2\lambda \alpha^{2}e^{-{\left(\frac{\alpha }{x}\right)}^{2}}}{x^{3}\left[1-e^{-{\left(\frac{\alpha }{x}\right)}^{2}}\right]\left\{1+{\left[1-e^{-{\left(\frac{\alpha }{x}\right)}^{2}}\right]}^{\lambda }\right\}}, \end{array}$$

$$\begin{array}{c} h\left(x;\varphi \right)=\frac{4\lambda \alpha^{2}e^{-{\left(\frac{\alpha }{x}\right)}^{2}}{\left[1-e^{-{\left(\frac{\alpha }{x}\right)}^{2}}\right]}^{\lambda -1}}{x^{3}\left\{1-{\left[1-e^{-{\left(\frac{\alpha }{x}\right)}^{2}}\right]}^{\lambda }\right\}\left\{1+{\left[1-e^{-{\left(\frac{\alpha }{x}\right)}^{2}}\right]}^{\lambda }\right\}} \end{array}$$

and

$$\begin{array}{c} H\left(x;\varphi \right)=-\log\left(2\right)-\lambda \log\left[1-e^{-{\left(\frac{\alpha }{x}\right)}^{2}}\right]+\log\left\{1+{\left[1-e^{-{\left(\frac{\alpha }{x}\right)}^{2}}\right]}^{\lambda }\right\},\;x,\lambda ,\alpha >0. \end{array}$$

Implications of these functions in survival analysis can be found in [31]. For modelling purposes, the pdf and hrf are informative on the ability of the HLIR model to fit data. For this reason, in the next, we put a focus on these two functions.

### 2.2. Functions Analysis

The limit features of the cdf, pdf and hrf of the HLIR distribution, i.e., given by Equation (4), Equation (5) and Equation (6), are studied below. In the case where x→0, we have

$$F\left(x;\varphi \right)∼\frac{\lambda }{2}e^{-{\left(\frac{\alpha }{x}\right)}^{2}},\;f\left(x;\varphi \right)∼\frac{\lambda \alpha^{2}}{x^{3}}e^{-{\left(\frac{\alpha }{x}\right)}^{2}},\;h\left(x;\varphi \right)∼\frac{\lambda \alpha^{2}}{x^{3}}e^{-{\left(\frac{\alpha }{x}\right)}^{2}}.$$

Thus, both the pdf and hrf tend to 0 in this case, with the same polyno-exponential decay. Also, we see that the rate of convergence mainly depends on the parameter α.

On the other side, when x→+∞, we get

$$F\left(x;\varphi \right)∼1-2{\left(\frac{\alpha }{x}\right)}^{2\lambda },\;f\left(x;\varphi \right)∼\frac{4\lambda }{x}{\left(\frac{\alpha }{x}\right)}^{2\lambda },\;h\left(x;\varphi \right)∼\frac{2\lambda }{x}.$$

Hence, in this case, both the pdf and hrf tend to 0. The rate of convergence of the pdf mainly depends on the parameter λ, whereas the one of the hrf is fixed and of order 1/x.

The mode(s) of the HLIR distribution is(are) given by critical point(s) of the corresponding pdf. Here, after some algebraic manipulations, it(they) is(are) given as solution(s) of the following non-linear equation:

$$\begin{array}{c} 2\alpha^{2}-3x^{2}-2\alpha^{2}\left(\lambda -1\right)\frac{e^{-{\left(\frac{\alpha }{x}\right)}^{2}}}{1-e^{-{\left(\frac{\alpha }{x}\right)}^{2}}}+4\lambda \alpha^{2}\frac{e^{-{\left(\frac{\alpha }{x}\right)}^{2}}{\left[1-e^{-{\left(\frac{\alpha }{x}\right)}^{2}}\right]}^{\lambda -1}}{1+{\left[1-e^{-{\left(\frac{\alpha }{x}\right)}^{2}}\right]}^{\lambda }}=0. \end{array}$$

Closed-form(s) for the mode(s) is(are) not available, but a mathematical software must help for a numerical evaluation.

On the other side, the critical point(s) is(are) given by the solution(s) of the following non-linear equation:

$$\begin{array}{c} 2\alpha^{2}-3x^{2}+2\alpha^{2}\frac{e^{-{\left(\frac{\alpha }{x}\right)}^{2}}}{1-e^{-{\left(\frac{\alpha }{x}\right)}^{2}}}+2\lambda \alpha^{2}\frac{e^{-{\left(\frac{\alpha }{x}\right)}^{2}}{\left[1-e^{-{\left(\frac{\alpha }{x}\right)}^{2}}\right]}^{\lambda -1}}{1+{\left[1-e^{-{\left(\frac{\alpha }{x}\right)}^{2}}\right]}^{\lambda }}=0. \end{array}$$

This(these) critical point(s) is(are) not expressible in an easy manner, but can be evaluated numerically.

**Figure 1:**
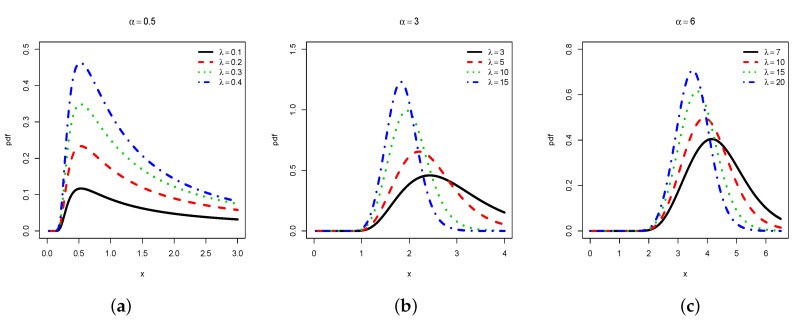
Plots of the pdf of the HLIR distribution for (**a**) α=0.5; (**b**) α=3 and (**c**) α=6, with varying λ.

**Figure 2:**
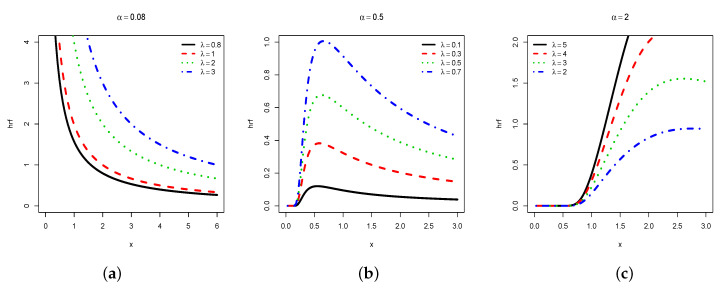
Plots of the hrf of the HLIR distribution for (**a**) α=0.08; (**b**) α=0.5 and (**c**) α=2, with varying λ.

A more direct analysis of the shapes comes from graphical investigations. In this regard, Figure 1 and Figure 2 plot the pdf and hrf, respectively, for selected values of φ.

We observe that the pdf of the HLIR distribution can be unimodal, and higlhy right-skewed (see Figure 1a), moderately right-skewed (see Figure 1b), or near symmetrical (see Figure 1c), with various heaviness on the tails. Moreover, we see that the hrf of the HLIR distribution can be decreasing with a reversed J-shape (see Figure 2a), increasing-decreasing with a reversed bathtub shape (see Figure 2b), or mainly increasing (see Figure 2c). In the curvature sense, these functions are significantly more flexible in comparison to those of the former IR distribution, motivating the consideration of the HLIR model for greatest statistical perspectives.

### 2.3. Quantile Function

The quantile function (qf) of the HLIR distribution, say Q(u;φ), can be obtained by inverting the corresponding cdf; it satisfies F[Q(u;φ);φ]=u for u∈(0,1). After some algebraic manipulations, we arrive at

(7) $$\begin{array}{c} Q\left(u;\varphi \right)=\alpha {\left[-\log\left(1-{\left(\frac{1-u}{1+u}\right)}^{\frac{1}{\lambda }}\right)\right]}^{-\frac{1}{2}},\;u\in \left(0,1\right). \end{array}$$

Thanks to its closed-form, this qf is helpful for determining the quartiles of the HLIR distribution, generating values from the HLIR distribution for simulation purposes and defining various measures of skewness and kurtosis. All these aspects will be used later.

## 3. Mathematical Properties

In this section, some notable mathematical properties of the HLIR distribution are derived, specifically stochastic ordering results, a general linear representation for the exponentiated probability density function, raw/inverted moments, incomplete moments, skewness and kurtosis features, and some entropy measures, namely, the Rényi entropy and *q*-entropy.

### 3.1. Some Stochastic Ordering Results

The HLIR distribution enjoys tractable stochastic ordering results involving the corresponding cdf. From a statistical point of view, such results allow a better comprehension of the roles of the parameters in the fitting ability of the HLIR model. The most notable of them are presented below.

**Proposition** **1.** *The following inequalities holds:*

- *For any $\alpha_{1}\geq \alpha_{2}>0$ and λ,x>0, we have $F\left(x;\alpha_{1},\lambda \right)\leq F\left(x;\alpha_{2},\lambda \right)$.*
- *For any $\lambda_{1}\geq \lambda_{2}>0$ and α,x>0, we have $F\left(x;\alpha ,\lambda_{2}\right)\leq F\left(x;\alpha ,\lambda_{1}\right)$.*

**Proof.**  The proof is based on monotonic arguments with respect to the parameters.

- After some algebraic manipulations, we get
  $$\begin{array}{c} \frac{\partial }{\partial \alpha }F\left(x;\varphi \right)=-\frac{4\alpha \lambda e^{-{\left(\frac{\alpha }{x}\right)}^{2}}{\left[1-e^{-{\left(\frac{\alpha }{x}\right)}^{2}}\right]}^{\lambda -1}}{x^{2}{\left\{1+{\left[1-e^{-{\left(\frac{\alpha }{x}\right)}^{2}}\right]}^{\lambda }\right\}}^{2}}<0, \end{array}$$
  implying that F(x;φ) is strictly decreasing with respect to α. Therefore, for  any $\alpha_{1}\geq \alpha_{2}>0$ and λ,x>0, we have $F\left(x;\alpha_{1},\lambda \right)\leq F\left(x;\alpha_{2},\lambda \right)$.
- With the same methodology, we have
  $$\begin{array}{c} \frac{\partial }{\partial \lambda }F\left(x;\varphi \right)=\frac{2{\left[1-e^{-{\left(\frac{\alpha }{x}\right)}^{2}}\right]}^{\lambda }\left\{-\log\left[1-e^{-{\left(\frac{\alpha }{x}\right)}^{2}}\right]\right\}}{{\left\{1+{\left[1-e^{-{\left(\frac{\alpha }{x}\right)}^{2}}\right]}^{\lambda }\right\}}^{2}}>0, \end{array}$$
  implying that F(x;φ) is strictly increasing with respect to λ. Therefore, for  any $\lambda_{1}\geq \lambda_{2}>0$ and α,x>0, we have $F\left(x;\alpha ,\lambda_{2}\right)\leq F\left(x;\alpha ,\lambda_{1}\right)$.

This ends the proof of Proposition 1. □

The following result shows a simple relation between the HLIR distribution and two other distributions, including the EIR distribution by [23].

**Proposition** **2.** *For any x,α,λ>0, the following inequalities holds:*

$$F_{**}\left(x;\varphi \right)\leq F\left(x;\varphi \right)\leq F_{*}\left(x;\varphi \right),$$

where $F_{*}\left(x;\varphi \right)$ and $F_{**}\left(x;\varphi \right)$ are two cdfs: $F_{*}\left(x;\varphi \right)=1-{\left[1-e^{-{\left(\frac{\alpha }{x}\right)}^{2}}\right]}^{\lambda }$ is the cdf of the EIR distribution and $F_{**}\left(x;\varphi \right)=\left[x^{2\lambda }/\left(x^{2\lambda }+\alpha^{2\lambda }\right)\right]F_{*}\left(x;\varphi \right)$ is a weighted version of it, which remains a valid cdf.

**Proof.**  The proof follows from the definition of F(x;φ), involving $F_{*}\left(x;\varphi \right)$ as numerator, and the following inequalities: since $e^{y}\geq 1+y$ for any y∈R,

$$1\leq 1+{\left[1-e^{-{\left(\frac{\alpha }{x}\right)}^{2}}\right]}^{\lambda }\leq 1+{\left(\frac{\alpha }{x}\right)}^{2\lambda }=\frac{x^{2\lambda }+\alpha^{2\lambda }}{x^{2\lambda }}.$$

This ends the proof of Proposition 2. □

In fact, the cdf $F_{**}\left(x;\varphi \right)$ in Proposition 2 is the cdf of the maximum of two independent random variables: one following the EIR distribution and the other following a special case of the power Lomax (PL) distribution introduced by [32].

### 3.2. Linear Representation

The following result introduces a useful linear representation for the exponentiated pdf of the HLIR distribution with power parameter ν>0.

**Proposition** **3.** *Let ν>0. Then, $f{\left(x;\varphi \right)}^{\nu }$ can be expressed as the following series expansion:*

$$\begin{array}{c} f{\left(x;\varphi \right)}^{\nu }=\sum_{j,k=0}^{+\infty }c_{j,k}\left(\varphi ,\nu \right)g_{k}\left(x;\varphi ,\nu \right), \end{array}$$

*where*

cj,k(φ,ν)=4νλνα2ν−2νjλj+ν(λ−1)k(−1)k,gk(x;φ,ν)=x−3νe−(k+ν)αx2,

*and ba denotes the generalized binomial coefficient.*

**Proof.**  The generalized binomial series formula applied two times in a row yields

f(x;φ)ν=4νλνα2νx−3νe−ναx21−e−αx2ν(λ−1)1+1−e−αx2λ−2ν=4νλνα2νx−3νe−ναx2∑j=0+∞−2νj1−e−αx2λj+ν(λ−1)=4νλνα2νx−3ν∑j,k=0+∞−2νjλj+ν(λ−1)k(−1)ke−(k+ν)αx2.

After a rearrangement, we get the desired result, ending the proof of Proposition 3. □

By taking ν=1 in Proposition 3, we get a useful series expansion for the pdf of the HLIR distribution, “useful” in the sense that we express a sophisticated function as sums of tractable functions, i.e., $g_{k}\left(x;\varphi ,\nu \right)$. In particular, we will use it in the next to provide measures and functions which are easy to handle from the analytical and numerical point of views.

### 3.3. Raw/Inverted Moments

Let *r* be an integer; the negative values are allowed. If *X* denotes a random variable following the HLIR distribution, then its $r^{th}$ moment (or (−r)^th^ inverted moment if *r* is negative) exists if and only if r<2λ, and it is given by $\mu_{r}^{\prime }=E\left(X^{r}\right)=\int_{0}^{+\infty }x^{r}f\left(x;\varphi \right)dx$. Thanks to Proposition 3 applied with ν=1 and the calculus of the integral $\int_{0}^{+\infty }x^{r}g_{k}\left(x;\varphi ,1\right)dx$ via the change of variable $y=\left(k+1\right){\left(\alpha /x\right)}^{2}$, assuming that r<2min(λ,1), we obtain the following relation

$$\begin{array}{c} \mu_{r}^{\prime }=\sum_{j,k=0}^{+\infty }c_{j,k}\left(\varphi ,1\right)\int_{0}^{+\infty }x^{r}g_{k}\left(x;\varphi ,1\right)dx=\sum_{j,k=0}^{+\infty }\frac{d_{j,k}\left(\varphi ,r\right)}{{\left(1+k\right)}^{1-\frac{r}{2}}}, \end{array}$$

where

dj,k(φ,r)=12αr−2Γ1−r2cj,k(φ,1)=2λαrΓ1−r2−2jλ(j+1)−1k(−1)k

and $\Gamma \left(s\right)=\int_{0}^{+\infty }x^{s-1}e^{-x}dx$ (the standard gamma function).

For instance, the mean of *X*, say μ, can be derived by taking r=1, and the following approximation remains acceptable:

$$\begin{array}{c} \mu =\mu_{1}^{\prime }=\sum_{j,k=0}^{+\infty }\frac{d_{j,k}\left(\varphi ,1\right)}{{\left(1+k\right)}^{\frac{1}{2}}}\approx \sum_{j,k=0}^{M}\frac{d_{j,k}\left(\varphi ,1\right)}{{\left(1+k\right)}^{\frac{1}{2}}}, \end{array}$$

where *M* denotes a large integer and

dj,k(φ,1)=2λαπ−2jλ(j+1)−1k(−1)k.

The variance of *X* does not exist, as well as the standard coefficients of skewness and kurtosis. This motivates the use of other measures of skewness and kurtosis based on quantiles, as performed in Section 3.5. However, we can express all the inverted moments, i.e., $\mu_{-1}^{\prime }$, $\mu_{-2}^{\prime }$, $\mu_{-3}^{\prime }$ and so on.

### 3.4. Incomplete Moments

Let *r* be an integer; the negative values are allowed. Contrary to the moments, the incomplete moments of the HLIR distribution always exist. That is, if *X* denotes a random variable following the HLIR distribution, for a given t≥0, the $r^{th}$ incomplete moment of *X* at *t* is given by $\mu_{r}^{\prime }\left(t\right)=E\left(X^{r}1_{\left\{X\leq t\right\}}\right)=\int_{0}^{t}x^{r}f\left(x;\varphi \right)dx$. Proceeding as for the raw/inverted moments, the following formula holds:

$$\begin{array}{c} \mu_{r}^{\prime }\left(t\right)=\sum_{j,k=0}^{+\infty }c_{j,k}\left(\varphi ,1\right)\int_{0}^{t}x^{r}g_{k}\left(x;\varphi ,1\right)dx=\sum_{j,k=0}^{+\infty }\frac{e_{j,k}\left(\varphi ,r\right)}{{\left(1+k\right)}^{1-\frac{r}{2}}}\Gamma \left(1-\frac{r}{2},\left(k+1\right){\left(\frac{\alpha }{t}\right)}^{2}\right), \end{array}$$

where

ej,k(φ,r)=12αr−2cj,k(φ,1)=2λαr−2jλ(j+1)−1k(−1)k

and $\Gamma \left(s,u\right)=\int_{u}^{+\infty }x^{s-1}e^{-x}dx$ (the upper incomplete gamma function).

We rediscover the relation $\lim_{t\to +\infty }\mu_{r}^{\prime }\left(t\right)=\mu_{r}^{\prime }$. Also, we can derive the $r^{th}$ normalized incomplete moment as $\phi_{r}\left(t\right)=\mu_{r}^{\prime }\left(t\right)/\mu_{r}^{\prime }$, which is key tool to define widely used measures of inequality, such as income quintiles, Lorenz curve, Pietra ratio and Gini coefficient. For instance, the Lorenz curve is the plot of $\left(\phi_{0}\left(t\right),\phi_{1}\left(t\right)\right)$ and the Gini coefficient is defined by

$$G=\int_{0}^{+\infty }\left[\frac{t}{\mu }\phi_{0}\left(t\right)-\phi_{1}\left(t\right)\right]f\left(t;\varphi \right)dt.$$

These measures are essential to go further with the HLIR distribution in applied settings. Further details and applications of them can be found in [33,34], and the references therein.

### 3.5. Skewness and Kurtosis Based on the qf

**Figure 3:**
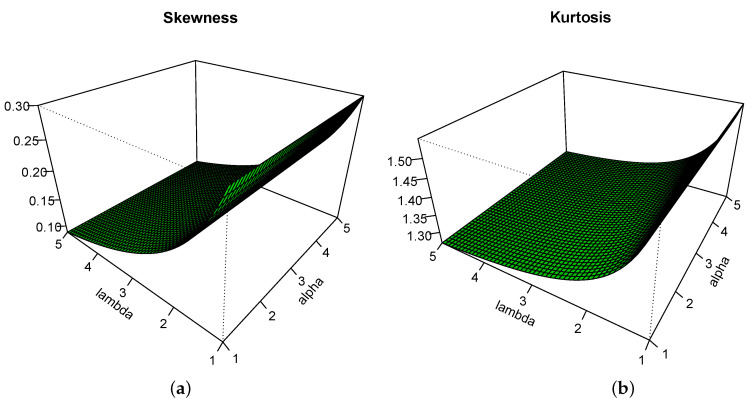
Plots for (**a**) the skewness *S* and (**b**) the kurtosis *K* for α,λ∈(1,5).

We now provide skewness and kurtosis analyzes of the HLIR distribution by using some measures involving the qf given by (7). Let us set $Q_{u}=Q\left(u;\varphi \right)$ with u∈(0,1). Then, we consider the coefficient of skewness *S* by [35] and the coefficient of kurtosis *K* by [36] defined by

$$S=\frac{Q_{3/4}-2Q_{1/2}+Q_{1/4}}{Q_{3/4}-Q_{1/4}},\;K=\frac{Q_{7/8}-Q_{5/8}+Q_{3/8}-Q_{1/8}}{Q_{3/4}-Q_{1/4}},$$

respectively. Here, *S* measures the degree of asymmetry of the HLIR distribution, whereas *K* measures the degree of its tail heaviness; as *K* increases, the tail of the HLIR distribution becomes heavier. To better handle these measures and see the effects of α and λ on them, Figure 3 displays the two-dimensional plots for *S* and *K* with respect to α and λ, with  α,λ∈(1,5).

From Figure 3, we see that the parameter α has a minor effect on *S* and *K*, contrary to λ; as λ increases, *S* and *K* increase. Also, we see that the HLIR distribution is mainly right-skewed, confirming the prime graphical investigations on the corresponding pdf.

### 3.6. Measures Of Entropy

The entropy of the HLIR distribution can be measured in different ways. Here, we focus our attention on the Rényi entropy by [37] and its twin sister: the *q*-entropy by [38]. For discussions and applications of these two entropy measures, we refer the reader to the survey of [39], and the references therein.

Let δ≠1 and δ>0. Then, when δ(2λ+1)>1, the Rényi entropy of the HLIR distribution exists, and it is given by

(8) $$\begin{array}{c} I_{\delta }\left(\varphi \right)=\frac{1}{1-\delta }\log\left[\int_{0}^{+\infty }f{\left(x;\varphi \right)}^{\delta }dx\right]. \end{array}$$

Let us now investigate a practical series expansion of the main term. Owing to Proposition 3 applied with ν=δ and the calculus of the integral $\int_{0}^{+\infty }g_{k}\left(x;\varphi ,\delta \right)dx$ via the change of variable $y=\left(k+\delta \right){\left(\alpha /x\right)}^{2}$, assuming that δ>max(1/(2λ+1),1/3), we get

(9) $$\begin{array}{c} \int_{0}^{+\infty }f{\left(x;\varphi \right)}^{\delta }dx=\sum_{j,k=0}^{+\infty }c_{j,k}\left(\varphi ,\delta \right)\int_{0}^{+\infty }g_{k}\left(x;\varphi ,\delta \right)dx=\sum_{j,k=0}^{+\infty }\frac{\ell_{j,k}\left(\varphi ,\delta \right)}{{\left(\delta +k\right)}^{\frac{3\delta }{2}-\frac{1}{2}}}, \end{array}$$

where

ℓj,k(φ,δ)=12a1−3δΓ3δ2−12cj,k(φ,δ)=22δ−1λδa1−δΓ3δ2−12−2δjλj+δ(λ−1)k(−1)k.

Therefore, one can express $I_{\delta }$, along with an acceptable approximation, as follows:

$$\begin{array}{c} I_{\delta }\left(\varphi \right)=\frac{1}{1-\delta }\log\left[\sum_{j,k=0}^{+\infty }\frac{\ell_{j,k}\left(\varphi ,\delta \right)}{{\left(\delta +k\right)}^{\frac{3\delta }{2}-\frac{1}{2}}}\right]\approx \frac{1}{1-\delta }\log\left[\sum_{j,k=0}^{M}\frac{\ell_{j,k}\left(\varphi ,\delta \right)}{{\left(\delta +k\right)}^{\frac{3\delta }{2}-\frac{1}{2}}}\right], \end{array}$$

where *M* denotes a large integer.

Now, let q≠1 and q>0. Then, the *q*-entropy of the HLIR distribution is defined by

(10) $$\begin{array}{c} H_{q}\left(\varphi \right)=\frac{1}{q-1}\left[1-\int_{0}^{+\infty }f{\left(x;\varphi \right)}^{q}dx\right]. \end{array}$$

Therefore, owing to (9), with a similar approach than above, we get

$$\begin{array}{c} H_{q}\left(\varphi \right)=\frac{1}{q-1}\left[1-\sum_{j,k=0}^{+\infty }\frac{\ell_{j,k}\left(\varphi ,q\right)}{{\left(q+k\right)}^{\frac{3q}{2}-\frac{1}{2}}}\right]\approx \frac{1}{q-1}\left[1-\sum_{j,k=0}^{M}\frac{\ell_{j,k}\left(\varphi ,q\right)}{{\left(q+k\right)}^{\frac{3q}{2}-\frac{1}{2}}}\right]. \end{array}$$

Other kinds of entropy can be expressed in a similar manner. In this regard, the book of [40] is suggested.

## 4. Estimation

By considering the HLIR distribution as a statistical model, this section investigates the estimation of α and λ via the five different methods mentioned in the introduction, also providing the interval estimation of these parameters, and the estimation of the Rényi entropy and *q*-entropy as well.

### 4.1. Estimation of The Parameters

A myriad of estimation methods can be used to estimate α and λ. Here, we focus on the most notable of them, namely the maximum likelihood (ML), least square (LS), weighted least square (WLS), percentile (PC) and Cramer-von Mises (CV) methods. We first describe their mathematical backgrounds and perform an adequate simulation study to check their efficiency.

#### 4.1.1. ML Method

First of all, we investigate the ML estimates (MLEs) of α and λ. Let $x_{1},\ldots ,x_{n}$ be *n* observed values from the HLIR distribution. Then, the MLEs can be calculated by maximizing the following function:

$$\begin{array}{cc} l(\varphi ) & =\sum_{i=1}^{n}\log\left[f\left(x_{i};\varphi \right)\right]\\ \; & =n\log\left(4\right)+n\log\left(\lambda \right)+2n\log\left(\alpha \right)-3\sum_{i=1}^{n}\log\left(x_{i}\right)+\left(\lambda -1\right)\sum_{i=1}^{n}\log\left[1-e^{-{\left(\frac{\alpha }{x_{i}}\right)}^{2}}\right]\\ \; & -2\sum_{i=1}^{n}\log\left\{1+{\left[1-e^{-{\left(\frac{\alpha }{x_{i}}\right)}^{2}}\right]}^{\lambda }\right\}, \end{array}$$

with respect to α and λ.

From the analytical point of view, the MLEs of α and λ are the solutions of two non-linear equations using the first partial derivatives of l(φ), i.e., 

$$\begin{array}{c} \frac{\partial l(\varphi )}{\partial \alpha }=\frac{2n}{\alpha }+2\alpha \left(\lambda -1\right)\sum_{i=1}^{n}\frac{e^{-{\left(\frac{\alpha }{x_{i}}\right)}^{2}}}{x_{i}^{2}\left[1-e^{-{\left(\frac{\alpha }{x_{i}}\right)}^{2}}\right]}-4\alpha \lambda \sum_{i=1}^{n}\frac{e^{-{\left(\frac{\alpha }{x_{i}}\right)}^{2}}{\left[1-e^{-{\left(\frac{\alpha }{x_{i}}\right)}^{2}}\right]}^{\lambda -1}}{x_{i}^{2}\left\{1+{\left[1-e^{-{\left(\frac{\alpha }{x_{i}}\right)}^{2}}\right]}^{\lambda }\right\}} \end{array}$$

and

$$\begin{array}{c} \frac{\partial l(\varphi )}{\partial \lambda }=\frac{n}{\lambda }+\sum_{i=1}^{n}\log\left[1-e^{-{\left(\frac{\alpha }{x_{i}}\right)}^{2}}\right]-2\sum_{i=1}^{n}\frac{{\left[1-e^{-{\left(\frac{\alpha }{x_{i}}\right)}^{2}}\right]}^{\lambda }\log\left[1-e^{-{\left(\frac{\alpha }{x_{i}}\right)}^{2}}\right]}{1+{\left[1-e^{-{\left(\frac{\alpha }{x_{i}}\right)}^{2}}\right]}^{\lambda }}. \end{array}$$

Then, equating ∂l(φ)/∂α and ∂l(φ)/∂β to zeros and solving them simultaneously with respect to α and λ, we obtain the MLEs (the same derivative approach can be developed in the next methods, but we omit it for the sake of conciseness). Under some regularity conditions, the random versions of the MLEs are known to be consistent, asymptotically normal, efficient and equivariant. Also, the formulas for the corresponding standard errors, asymptotic confidence intervals and likelihood ratio tests involving the MLEs are well-known. In this regard, we may refer to [41]. From the practical point of view, they can be determine numerically thanks to the use of any statistical software (R, SAS, Python, MATHCAD…).

#### 4.1.2. Ordinary and Weighted LS Methods

Let $x_{1},\ldots ,x_{n}$ be *n* observed values from the HLIR distribution and $x_{\left(1\right)},x_{\left(2\right)},\ldots ,x_{\left(n\right)}$ be their ordered values, i.e., $x_{\left(1\right)}=\inf\left(x_{1},\ldots ,x_{n}\right)$, $x_{\left(2\right)}=\inf\left(\left\{x_{1},\ldots ,x_{n}\right\}\left\{x_{\left(1\right)}\right\}\right)$ …and $x_{n}=\sup\left(x_{1},\ldots ,x_{n}\right)$. Then, the LS estimates (LSEs) of α and λ can be calculated by minimizing the following function:

$$\begin{array}{c} LS\left(\varphi \right)=\sum_{i=1}^{n}{\left[F\left(x_{\left(i\right)};\varphi \right)-\frac{i}{n+1}\right]}^{2}=\sum_{i=1}^{n}{\left\{\frac{1-{\left[1-e^{-{\left(\frac{\alpha }{x_{\left(i\right)}}\right)}^{2}}\right]}^{\lambda }}{1+{\left[1-e^{-{\left(\frac{\alpha }{x_{\left(i\right)}}\right)}^{2}}\right]}^{\lambda }}-\frac{i}{n+1}\right\}}^{2}, \end{array}$$

with respect to α and λ.

Similarly, by introducing a thorough weighted sequence, the WLS estimates (WLSEs) of α and λ can be obtained by minimizing the following function:

$$\begin{array}{cc} WLS(\varphi ) & =\sum_{i=1}^{n}\frac{{\left(n+1\right)}^{2}\left(n+2\right)}{i\left(n-i+1\right)}{\left[F\left(x_{\left(i\right)};\varphi \right)-\frac{i}{n+1}\right]}^{2}\\ \; & =\sum_{i=1}^{n}{\frac{{\left(n+1\right)}^{2}\left(n+2\right)}{i\left(n-i+1\right)}\left\{\frac{1-{\left[1-e^{-{\left(\frac{\alpha }{x_{\left(i\right)}}\right)}^{2}}\right]}^{\lambda }}{1+{\left[1-e^{-{\left(\frac{\alpha }{x_{\left(i\right)}}\right)}^{2}}\right]}^{\lambda }}-\frac{i}{n+1}\right\}}^{2}, \end{array}$$

with respect to α and λ.

For the theoretical background of this method and applications as well, we refer the reader to [42].

#### 4.1.3. PC Method

Let $x_{1},\ldots ,x_{n}$ be *n* observed values from the HLIR distribution and $x_{\left(1\right)},x_{\left(2\right)},\ldots ,x_{\left(n\right)}$ be their ordered values. Then, the PC estimates (PCEs) of α and λ are derived by minimizing the following function:

$$\begin{array}{c} PC\left(\varphi \right)=\sum_{i=1}^{n}{\left[x_{\left(i\right)}-Q\left(\frac{i}{n+1};\varphi \right)\right]}^{2}=\sum_{i=1}^{n}{\left\{x_{\left(i\right)}-\alpha {\left[-\log\left(1-{\left(\frac{1-i/(n+1)}{1+i/(n+1)}\right)}^{\frac{1}{\lambda }}\right)\right]}^{-\frac{1}{2}}\right\}}^{2}, \end{array}$$

with respect to α and λ.

The basics of this method can be found in [43,44].

#### 4.1.4. CV Method

Let $x_{1},\ldots ,x_{n}$ be *n* observed values from the HLIR distribution and $x_{\left(1\right)},x_{\left(2\right)},\ldots ,x_{\left(n\right)}$ be their ordered values. The CV estimates (CVEs) is a type of minimum distance estimates which is based on the difference between the estimated and empirical cdfs (see [45,46]). That is, the CVEs of α and λ are obtained by minimizing the following function:

$$\begin{array}{cc} CV(\varphi ) & =\frac{1}{12n}+\sum_{i=1}^{n}{\frac{{\left(n+1\right)}^{2}\left(n+2\right)}{i\left(n-i+1\right)}\left[F\left(x_{\left(i\right)};\varphi \right)-\frac{2i-1}{2n}\right]}^{2}\\ \; & =\frac{1}{12n}+\sum_{i=1}^{n}{\frac{{\left(n+1\right)}^{2}\left(n+2\right)}{i\left(n-i+1\right)}\left[\frac{1-{\left[1-e^{-{\left(\frac{\alpha }{x_{\left(i\right)}}\right)}^{2}}\right]}^{\lambda }}{1+{\left[1-e^{-{\left(\frac{\alpha }{x_{\left(i\right)}}\right)}^{2}}\right]}^{\lambda }}-\frac{2i-1}{2n}\right]}^{2}, \end{array}$$

with respect to α and λ. Last but not least, we refer to [47] for empirical evidence of the efficiency of the CVEs.

#### 4.1.5. Numerical Results

Here, we conduct a simulation study to evaluate and compare the efficiency of the above estimates, with respect to their mean squared errors (MSEs). In this regard, for each n=10, 20, 30, 50, 100 and 200, we generate 1000 random samples of size *n*, i.e., of the form $\left(x_{1},x_{2},\ldots ,x_{n}\right)$, from the HLIR distribution (the inversion method involving the qf given by (7) is used). The four following different sets of parameters are considered:

φ1: (α=1.5, λ=0.8), φ2: (α=1.5,λ=0.5), φ3: (α=1.5,λ=1), φ4: (α=0.5,λ=0.5).

Then, the ML, LS, WLS, PC and CV estimates of α and λ are computed, as well as their MSEs. Simulated outcomes are listed in Table 1, Table 2, Table 3 and Table 4 for φ1, φ2, φ3 and φ4, respectively. The statistical software MATHCAD(14) is used.

From these tables, it is clear that the MSEs decrease as sample sizes increase, for all the estimates. Also, in almost all of the cases, the MSEs of the MLEs take the smallest values among the corresponding MSEs of the other methods. In this sense, in our setting, the ML method can be considered as the best and will be naturally privileged in the next.

We complete this part with an interval estimation study. More specifically, we use the ML method to calculate lower bound (LB), upper bound (UB) and average length (AL) of the (two-sided asymptotic) confidence interval estimation of the model parameters at the levels 90% and 95%. The obtained numerical results are mentioned in Table 5, Table 6, Table 7 and Table 8 for φ1, φ2, φ3 and φ4, respectively.

From Table 5, Table 6, Table 7 and Table 8, it is clear that the ALs decrease as sample sizes increase.

### 4.2. Estimation of The Entropy

We now investigate the estimation of the Rényi entropy $I_{\delta }\left(\varphi \right)$ given by (8) and the *q*-entropy $H_{q}\left(\varphi \right)$ given by (10) by the means of a simulation study. We adopt the same setting than Section 4.1.5 (we generate 1000 random samples of sizes n=10, 20…with the same sets of parameters). Then, we determine the MLEs of α and λ, denoted by $\hat{\alpha }$ and $\hat{\lambda }$, and for δ,q=1.2, 1.5 and 2, we estimate $I_{\delta }\left(\varphi \right)$ and $H_{q}\left(\varphi \right)$ by $I_{\delta }\left(\hat{\varphi }\right)$ and $H_{q}\left(\hat{\varphi }\right)$ with $\hat{\varphi }=\left(\hat{\alpha },\hat{\lambda }\right)$, respectively. We measure the precision of these estimates by the relative biases (RBs); in the case of the Rényi entropy and a given φ, the RB is defined by

$$RB=\frac{\mathrm{Estimate}\;I_{\delta }\left(\hat{\varphi }\right)-\mathrm{Exact}\;\mathrm{value}\;I_{\delta }\left(\varphi \right)}{\mathrm{Exact}\;\mathrm{value}\;I_{\delta }\left(\varphi \right)}.$$

Simulated outcomes about the Rényi entropy are presented in Table 9, Table 10, Table 11 and Table 12 for φ1, φ2, φ3 and φ4, respectively, and those of the *q*-entropy are presented in Table 13, Table 14, Table 15 and Table 16 for φ1, φ2, φ3 and φ4, respectively.

From these tables, we see that the RBs of the Rényi entropy and *q*-entropy decrease as the sample sizes increase, attesting the efficiency of the proposed estimates.

## 5. Applications to Real Data

In this section, we prove the flexibility of the HLIR model by analyzing two practical datasets, denoted by D1 and D2, given as follows:

D1. The first data set is extracted from ([48], Table 3). For the sake of transparency, the data are: 1.312, 1.314, 1.479, 1.552, 1.700, 1.803, 1.861, 1.865, 1.944, 1.958, 1.966, 1.997, 2.006, 2.021, 2.027, 2.055, 2.063, 2.098, 2.14, 2.179, 2.224, 2.240, 2.253, 2.270, 2.272, 2.274, 2.301, 2.301, 2.359, 2.382, 2.382, 2.426, 2.434, 2.435, 2.478, 2.490, 2.511, 2.514, 2.535, 2.554, 2.566, 2.57, 2.586, 2.629, 2.633, 2.642, 2.648, 2.684, 2.697, 2.726, 2.770, 2.773, 2.800, 2.809, 2.818, 2.821, 2.848, 2.88, 2.954, 3.012, 3.067, 3.084, 3.090, 3.096, 3.128, 3.233, 3.433, 3.585, 3.585.
D2. The second data set coming from [49]. The data consists of the monthly actual taxes revenue in Egypt from January 2006 to November 2010. The data (in 1000 million Egyptian pounds) are: 5.9, 20.4, 14.9, 16.2, 17.2, 7.8, 6.1, 9.2, 10.2, 9.6, 13.3, 8.5, 21.6, 18.5, 5.1, 6.7, 17, 8.6, 9.7, 39.2, 35.7, 15.7, 9.7, 10, 4.1, 36, 8.5, 8, 9.2, 26.2, 21.9, 16.7, 21.3, 35.4, 14.3, 8.5, 10.6, 19.1, 20.5, 7.1, 7.7, 18.1, 16.5, 11.9, 7, 8.6, 12.5, 10.3, 11.2, 6.1, 8.4, 11, 11.6, 11.9, 5.2, 6.8, 8.9, 7.1, 10.8. The corresponding histogram shows that the distribution of the data is highly right-skewed, motivating the use of the HLIR model for suitable fits.

The fitting behavior of the HLIR model is compared to the one of the following well-known models: OFIR, TIITLIR, TIR and IR models. As common point, they extend the IR model and possess two parameters (excepted the former IR model). With the concern to make comparisons as fair as possible, we apply the following notorious criteria: Cramér Von-Mises (CVM), Anderson-Darling (AD), Kolmogorov-Smirnov (KS) with the corresponding p-value (KS p-value), as well as those based on the log-likelihood: minus estimated log-likelihood (-$\hat{\ell }$), Akaike information criterion (AIC), consistent Akaike information criterion (CAIC), Bayesian information criterion (BIC) and Hannan-Quinn information criterion (HQIC). Model with the minimum values for CVM, AD, KS, (-$\hat{\ell }$), AIC, BIC, CAIC and HQIC, and the maximum KS p-value, is considered to provide the best fits for the proposed data. For further details on these criteria, we refer the reader to [50]. In this part, the software R is used, along with the package AdequacyModel (see [51]).

The MLEs and their standard errors (SEs) for the considered models, as well as CVM, AD, KS, and KS p-value are given in Table 17 and Table 18 for D1 and D2, respectively. Also, the goodness-of-fit measures $\left(-\hat{\ell }\right)$, AIC, BIC, CAIC, and HQIC are provided in Table 19 and Table 20 for D1 and D2, respectively.

From Table 17, Table 18, Table 19 and Table 20, we confirm that the HLIR model provides the best fits among the other models for D1 and D2 since it has the lowest values of CVM, AD, KS, $-\hat{\ell }$, AIC, CAIC, BIC and HQIC, and the greatest values for the KS p-value. Also, one can notice that the KS p-values of the HLIR model are very closed to 1, making the HLIR model difficult to beat with the KS p-value benchmark for D1 and D2.

**Figure 4:**
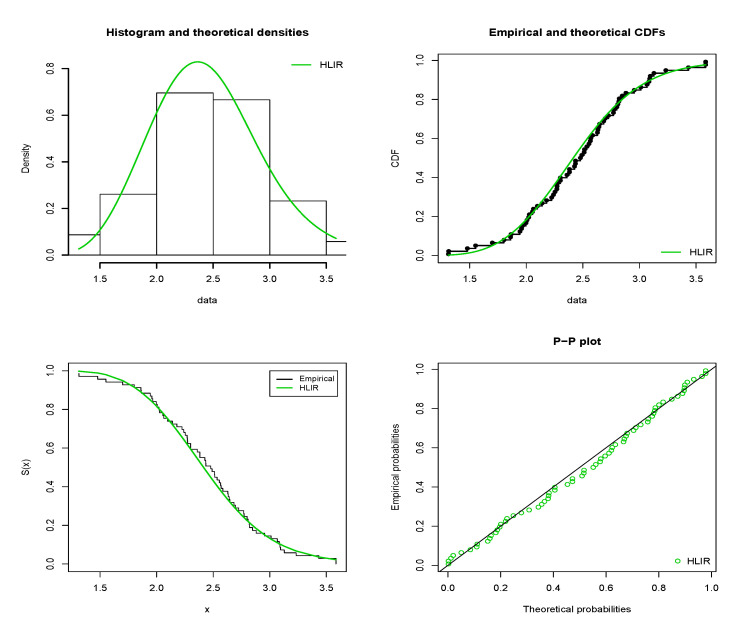
Plots for the epdf, ecdf, esf and P-P plots of the HLIR model for D1.

**Figure 5:**
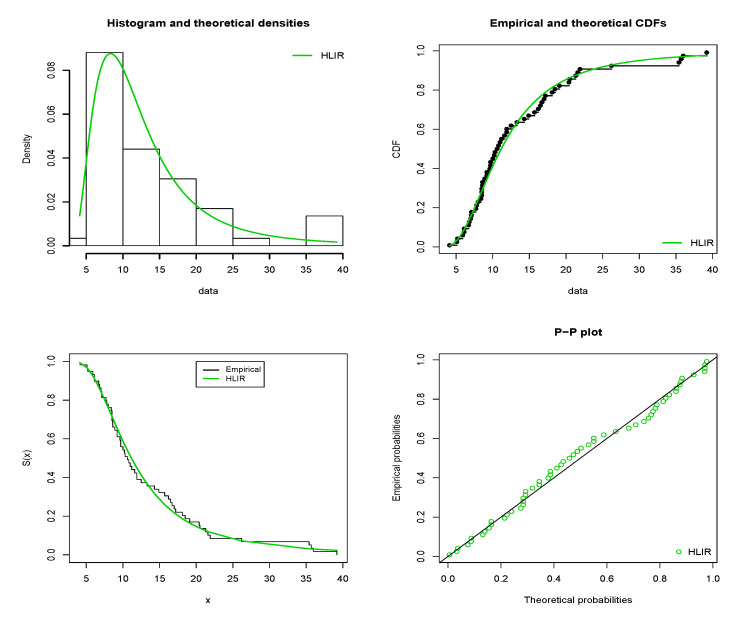
Plots for the epdf, ecdf, esf and P-P plots of the HLIR model for D2.

The estimated pdf (epdf), estimated cdf (ecdf), estimated sf (esf) and probability-probability (P-P) plots of the HLIR model are displayed in Figure 4 and Figure 5 for D1 and D2, respectively.

From Figure 4 and Figure 5, nice fits are observed for the HLIR models; in the four plots, the green curves fit very well those based on the corresponding empirical ones, attesting the applicability of the HLIR model for D1 and D2.

In order to complete this part, let us now investigate the entropy estimation of the HLIR model for the two data sets, adopting the methodology describes in Section 3.6. For D1, at δ=1.2, δ=1.5, and δ=2, the estimated Rényi entropy is equal to 49.182, 25.324 and 17.371, respectively, and, at q=1.2, q=1.5 and q=2, the estimated *q*-entropy is equal to 5, 2 and 1, respectively. Thus, we can notice that the estimated Rényi entropy decreases as δ increases. Also, the estimated *q*-entropy decreases as *q* increases. For D2, at δ=1.2, δ=1.5 and δ=2, the estimated Rényi entropy is equal to 1.348, 1.304 and 1.259, respectively, and, at q=1.2, q=1.5 and q=2, the estimated *q*-entropy is equal to 2.312, 1.555 and 0.945, respectively. Hence, the estimated Rényi entropy decreases as δ increases. Also, the estimated *q*-entropy decreases as *q* increases.

## 6. Concluding Remarks

In this paper, a new two-parameter lifetime distribution based on the half-logistic transformation and the IR distribution is introduced. It is called the HLIR distribution. Some of its mathematical properties as stochastic ordering results, a general linear representation for the exponentiated probability density function, raw/inverted moments, incomplete moments, skewness and kurtosis features, and Rényi entropy and *q*-entropy are derived. The estimation of the model parameters is discussed through the ML, LS, WLS, PC and CV methods. Simulation study is carried out to compare the performance of the five resulting estimates. It revealed that the ML method performs better than the others, in approximately, most of situations. The estimation of the Rényi entropy and *q*-entropy is also conducted with success. An application to two real data sets indicates that the HLIR model can produce better fits than other champion models, also based on the IR distribution. With only two parameters and such a high degree of performance, we hope that the HLIR model will attract the attention of some practitioners for further perspectives.

## Figures and Tables

**Table 1:** Estimates and MSEs with the ML, LS, WLS, PC and CV methods for the HLIR model with φ1.

*n*	MLEs		LSEs		WLSEs		PCEs		CVEs	
	Es	MSE	Es	MSE	Es	MSE	Es	MSE	Es	MSE
10	1.713	0.302	1.478	0.374	1.471	0.287	1.378	0.319	1.744	1.379
	0.991	0.287	0.855	0.447	0.841	0.395	1.005	7.810	1.109	1.215
20	1.627	0.130	1.496	0.142	1.511	0.126	1.402	0.142	1.623	0.843
	0.904	0.079	0.828	0.078	0.835	0.071	0.798	0.145	0.928	0.130
30	1.567	0.065	1.492	0.090	1.510	0.082	1.373	0.098	1.575	0.699
	0.856	0.034	0.808	0.043	0.818	0.040	0.745	0.075	0.866	0.059
50	1.545	0.037	1.502	0.054	1.514	0.045	1.408	0.057	1.553	0.623
	0.838	0.020	0.814	0.026	0.819	0.022	0.752	0.045	0.849	0.032
100	1.523	0.018	1.503	0.026	1.513	0.022	1.424	0.031	1.527	0.555
	0.817	8.129 *	0.805	0.012	0.810	9.531 *	0.747	0.024	0.821	0.013
200	1.507	8.193 *	1.497	8.467 *	1.500	9.781 *	1.446	0.017	1.506	0.511
	0.807	4.213 *	0.800	3.667 *	0.803	4.774 *	0.761	0.014	0.808	5.512 *

The symbol * indicate that the value multiply $10^{-3}$.

**Table 2:** Estimates and MSEs with the ML, LS, WLS, PC and CV methods for the HLIR model with φ2.

*n*	MLEs		LSEs		WLSEs		PCEs		CVEs	
	Es	MSE	Es	MSE	Es	MSE	Es	MSE	Es	MSE
10	1.822	0.613	1.521	0.617	1.510	0.542	1.398	0.455	1.868	2.750
	0.615	0.088	0.532	0.087	0.534	0.117	0.555	0.275	0.661	0.199
20	1.682	0.211	1.508	0.235	1.559	0.243	1.407	0.196	1.673	1.673
	0.554	0.021	0.508	0.023	0.525	0.030	0.498	0.044	0.560	0.035
30	1.590	0.104	1.486	0.151	1.511	0.121	1.378	0.131	1.591	1.360
	0.536	0.014	0.506	0.014	0.514	0.014	0.485	0.032	0.539	0.019
50	1.557	0.057	1.485	0.074	1.511	0.070	1.395	0.074	1.547	1.176
	0.517	6.076 *	0.499	7.249 *	0.504	6.665 *	0.473	0.014	0.517	8.380 *
100	1.522	0.025	1.504	0.038	1.500	0.030	1.420	0.041	1.534	1.109
	0.507	2.733 *	0.501	3.607 *	0.502	2.987 *	0.476	7.316 *	0.510	3.905 *
200	1.516	0.011	1.503	0.021	1.508	0.014	1.445	0.020	1.518	1.058
	0.504	1.175 *	0.500	1.744 *	0.502	1.370 *	0.479	4.204 *	0.505	1.811 *

The symbol * indicates that the value multiply $10^{-3}$.

**Table 3:** Estimates and MSEs with the ML, LS, WLS, PC and CV methods for the HLIR model with φ3.

*n*	MLEs		LSEs		WLSEs		PCEs		CVEs	
	Es	MSE	Es	MSE	Es	MSE	Es	MSE	Es	MSE
10	1.696	0.263	1.466	0.270	1.477	0.248	1.383	0.272	1.706	0.842
	1.314	0.705	1.128	1.309	1.132	1.397	1.233	5.499	1.496	3.430
20	1.616	0.102	1.513	0.131	1.526	0.114	1.408	0.129	1.630	0.545
	1.137	0.124	1.082	0.606	1.07	0.217	1.003	0.345	1.233	1.352
30	1.556	0.055	1.485	0.072	1.499	0.062	1.388	0.092	1.56	0.391
	1.086	0.074	1.025	0.085	1.033	0.073	0.965	0.295	1.107	0.119
50	1.537	0.032	1.489	0.045	1.502	0.037	1.406	0.054	1.533	0.332
	1.043	0.033	1.002	0.043	1.012	0.036	0.929	0.079	1.047	0.052
100	1.513	0.014	1.490	0.020	1.498	0.016	1.429	0.030	1.522	0.295
	1.017	0.014	0.999	0.019	1.005	0.016	0.938	0.044	1.027	0.022
200	1.511	6.475 *	1.500	0.010	1.505	7.969 *	1.452	0.014	1.511	0.271
	1.009	5.961 *	1.000	8.878 *	1.005	7.127 *	0.947	0.024	1.011	9.286 *

The symbol * indicates that the value multiply $10^{-3}$.

**Table 4:** Estimates and MSEs with the ML, LS, WLS, PC and CV methods for the HLIR model with φ4.

*n*	MLEs		LSEs		WLSEs		PCEs		CVEs	
	Es	MSE	Es	MSE	Es	MSE	Es	MSE	Es	MSE
10	0.606	0.069	0.502	0.069	0.506	0.063	0.466	0.051	0.621	0.126
	0.615	0.088	0.538	0.145	0.541	0.143	0.557	0.284	0.664	0.213
20	0.561	0.023	0.515	0.031	0.520	0.027	0.469	0.022	0.554	0.032
	0.554	0.021	0.526	0.052	0.525	0.030	0.498	0.044	0.553	0.035
30	0.530	0.012	0.498	0.016	0.504	0.013	0.459	0.015	0.542	0.020
	0.536	0.014	0.510	0.015	0.514	0.014	0.485	0.032	0.540	0.017
50	0.519	6.332 *	0.498	9.623 *	0.504	7.787 *	0.465	8.251 *	0.521	0.011
	0.517	6.076 *	0.499	7.766 *	0.504	6.665 *	0.473	0.014	0.523	9.412 *
100	0.507	2.773 *	0.497	4.192 *	0.500	3.278 *	0.473	4.530 *	0.511	4.964 *
	0.507	2.733 *	0.499	3.481 *	0.502	2.987 *	0.476	7.316 *	0.512	4.168 *
200	0.505	1.222 *	0.501	2.110 *	0.503	1.600 *	0.482	2.182 *	0.504	1.996 *
	0.504	1.175 *	0.500	1.665 *	0.502	1.371 *	0.479	4.204 *	0.504	1.788 *

The symbol * indicates that the value multiply $10^{-3}$.

**Table 5:** LBs, UBs and ALs of the confidence interval estimation with the ML method for the HLIR model with φ1.

*n*	90%			95%		
	LB	UB	AL	LB	UB	AL
10	0.9812	2.3595	1.3783	0.8493	2.4915	1.6422
	0.4155	1.8072	1.3916	0.2823	1.9404	1.6581
20	1.0237	1.9731	0.9494	0.9328	2.0640	1.1311
	0.4570	1.0406	0.5835	0.4012	1.0964	0.6953
30	1.1899	1.9927	0.8028	1.1131	2.0696	0.9565
	0.5637	1.1046	0.5409	0.5119	1.1564	0.6445
50	1.3385	1.9514	0.6130	1.2798	2.0101	0.7303
	0.7090	1.2018	0.4928	0.6618	1.2490	0.5872
100	1.2681	1.6878	0.4197	1.2280	1.7280	0.5000
	0.6276	0.8917	0.2641	0.6023	0.9170	0.3147
200	1.3499	1.6483	0.2983	1.3214	1.6768	0.3554
	0.6857	0.8793	0.1936	0.6671	0.8978	0.2307

**Table 6:** LBs, UBs and ALs of the confidence interval estimation with the ML method for the HLIR model with φ2.

*n*	90%			95%		
	LB	UB	AL	LB	UB	AL
10	0.8230	2.4433	1.6203	0.6679	2.5984	1.9305
	0.2930	0.9958	0.7028	0.2257	1.0631	0.8374
20	1.1324	2.4175	1.2851	1.0093	2.5405	1.5312
	0.3437	0.7453	0.4016	0.3053	0.7838	0.4785
30	1.2235	2.2379	1.0144	1.1264	2.3351	1.2086
	0.3834	0.7141	0.3307	0.3517	0.7458	0.3941
50	1.1789	1.8935	0.7146	1.1105	1.9619	0.8515
	0.3984	0.6401	0.2416	0.3753	0.6632	0.2879
100	1.2926	1.8030	0.5104	1.2437	1.8519	0.6082
	0.4320	0.6013	0.1693	0.4158	0.6175	0.2017
200	1.3099	1.6611	0.3511	1.2763	1.6947	0.4184
	0.4385	0.5523	0.1138	0.4276	0.5632	0.1356

**Table 7:** LBs, UBs and ALs of the confidence interval estimation with the ML method for the HLIR model with φ3.

*n*	90%			95%		
	LB	UB	AL	LB	UB	AL
10	1.0988	2.3947	1.2959	0.9747	2.5187	1.5440
	0.5174	2.3797	1.8624	0.3390	2.5580	2.2190
20	1.2117	2.1173	0.9057	1.1249	2.2040	1.0791
	0.7164	1.8340	1.1176	0.6094	1.9410	1.3316
30	1.3016	2.0438	0.7422	1.2305	2.1149	0.8844
	0.7916	1.6449	0.8533	0.7099	1.7265	1.0167
50	1.3957	1.9756	0.5800	1.3401	2.0312	0.6910
	0.9009	1.5806	0.6797	0.8358	1.6457	0.8099
100	1.3614	1.7592	0.3978	1.3233	1.7973	0.4740
	0.8509	1.2381	0.3872	0.8138	1.2752	0.4614
200	1.3840	1.6621	0.2780	1.3574	1.6887	0.3313
	0.8758	1.1375	0.2618	0.8507	1.1626	0.3119

**Table 8:** LBs, UBs and ALs of the confidence interval estimation with the ML method for the HLIR model with φ4.

*n*	90%			95%		
	LB	UB	AL	LB	UB	AL
10	0.3509	0.9691	0.6182	0.2917	1.0283	0.7365
	0.3310	1.1897	0.8587	0.2488	1.2719	1.0232
20	0.3634	0.7614	0.3980	0.3253	0.7995	0.4742
	0.3625	0.7954	0.4329	0.3211	0.8368	0.5157
30	0.4275	0.7659	0.3384	0.3951	0.7983	0.4032
	0.4320	0.8254	0.3935	0.3943	0.8631	0.4688
50	0.3565	0.5830	0.2264	0.3349	0.6046	0.2698
	0.3658	0.5833	0.2174	0.3450	0.6041	0.2591
100	0.4149	0.5828	0.1680	0.3988	0.5989	0.2001
	0.4147	0.5764	0.1617	0.3993	0.5919	0.1926
200	0.4638	0.5858	0.1220	0.4521	0.5974	0.1454
	0.4591	0.5795	0.1204	0.4475	0.5911	0.1435

**Table 9:** Rényi entropy estimates and RBs for the HLIR model with φ1.

*n*	Exact Value	δ=1.2		Exact Value	δ=1.5		Exact Value	δ=2	
		Estimate	RB		Estimate	RB		Estimate	RB
10	0.929	0.836	0.100	0.858	0.743	0.134	0.787	0.708	0.101
20		0.908	0.023		0.829	0.034		0.770	0.022
30		0.910	0.020		0.832	0.031		0.779	0.011
50		0.917	0.013		0.842	0.018		0.782	6.454 *
100		0.937	8.576 *		0.854	4.477 *		0.786	1.323 *
200		0.929	0.252 *		0.858	0.004 *		0.788	0.968 *

The symbol * indicates that the value multiply $10^{-3}$.

**Table 10:** Rényi entropy estimates and RBs for the HLIR model with φ2.

*n*	Exact Value	δ=1.2		Exact Value	δ=1.5		Exact Value	δ=2	
		Estimate	RB		Estimate	RB		Estimate	RB
10	1.279	1.213	0.052	1.167	1.077	0.077	1.062	1.001	0.058
20		1.223	0.044		1.144	0.020		1.036	0.024
30		1.265	0.011		1.149	0.015		1.038	0.022
50		1.265	0.011		1.160	6.064 *		1.055	6.444 *
100		1.267	9.558 *		1.161	5.065 *		1.058	3.544 *
200		1.273	5.068 *		1.164	2.707 *		1.059	2.975 *

The symbol * indicates that the value multiply $10^{-3}$.

**Table 11:** Rényi entropy estimates and RBs for the HLIR model with φ3.

*n*	Exact Value	δ=1.2		Exact Value	δ=1.5		Exact Value	δ=2	
		Estimate	RB		Estimate	RB		Estimate	RB
10	0.789	0.668	0.153	0.73	0.640	0.123	0.671	0.610	0.091
20		0.714	0.095		0.705	0.035		0.637	0.050
30		0.761	0.035		0.712	0.026		0.659	0.017
50		0.776	0.016		0.713	0.025		0.663	0.012
100		0.779	0.012		0.722	0.011		0.669	3.100 *
200		0.788	1.497 *		0.725	6.639 *		0.671	0.357 *

The symbol * indicates that the value multiply $10^{-3}$.

**Table 12:** Rényi entropy estimates and RBs for the HLIR model with φ4.

*n*	Exact Value	δ=1.2		Exact Value	δ=1.5		Exact Value	δ=2	
		Estimate	RB		Estimate	RB		Estimate	RB
10	0.802	0.730	0.089	0.69	0.604	0.125	0.585	0.522	0.108
20		0.778	0.030		0.661	0.042		0.568	0.029
30		0.791	0.014		0.669	0.031		0.576	0.016
50		0.796	7.181 *		0.679	0.015		0.577	0.014
100		0.797	5.891 *		0.688	2.132 *		0.589	7.532 *
200		0.800	2.364 *		0.689	0.606 *		0.585	0.511 *

The symbol * indicates that the value multiply $10^{-3}$.

**Table 13:** *q*-entropy estimates and RBs for the HLIR model with φ1.

*n*	Exact Value	q=1.2		Exact Value	q=1.5		Exact Value	q=2	
		Estimate	RB		Estimate	RB		Estimate	RB
10	1.74	1.584	0.090	1.255	1.184	0.057	0.837	0.812	0.030
20		1.665	0.043		1.240	0.012		0.817	0.024
30		1.714	0.015		1.242	0.011		0.827	0.012
50		1.722	0.010		1.247	6.763 *		0.834	3.921 *
100		1.733	4.096 *		1.262	5.454 *		0.838	0.951 *
200		1.738	1.241 *		1.255	0.119 *		0.837	0.192 *

The symbol * indicates that the value multiply $10^{-3}$.

**Table 14:** *q*-entropy estimates and RBs for the HLIR model with φ2.

*n*	Exact Value	q=1.2		Exact Value	q=1.5		Exact Value	q=2	
		Estimate	RB		Estimate	RB		Estimate	RB
10	2.226	2.141	0.038	1.478	1.447	0.021	0.913	0.904	0.010
20		2.166	0.027		1.456	0.015		0.906	8.183 *
30		2.175	0.023		1.471	4.813 *		0.913	0.362 *
50		2.204	9.500 *		1.472	4.358 *		0.913	0.171 *
100		2.217	3.877 *		1.473	3.469 *		0.913	0.131 *
200		2.218	3.458 *		1.475	2.095 *		0.913	0.111 *

The symbol * indicates that the value multiply $10^{-3}$.

**Table 15:** *q*-entropy estimates and RBs for the HLIR model with φ3.

*n*	Exact Value	q=1.2		Exact Value	q=1.5		Exact Value	q=2	
		Estimate	RB		Estimate	RB		Estimate	RB
10	1.523	1.374	0.098	1.137	1.024	0.099	0.787	0.754	0.041
20		1.465	0.038		1.068	0.061		0.761	0.033
30		1.484	0.026		1.113	0.021		0.779	9.278 *
50		1.499	0.016		1.126	9.813 *		0.784	3.558 *
100		1.512	7.609 *		1.129	7.297 *		0.788	1.975 *
200		1.521	1.275 *		1.136	0.757 *		0.786	0.939 *

The symbol * indicates that the value multiply $10^{-3}$.

**Table 16:** *q*-entropy estimates and RBs for the HLIR model with φ4.

*n*	Exact Value	q=1.2		Exact Value	q=1.5		Exact Value	q=2	
		Estimate	RB		Estimate	RB		Estimate	RB
10	1.544	1.472	0.047	1.096	1.039	0.053	0.74	0.728	0.016
20		1.476	0.044		1.082	0.013		0.730	0.014
30		1.518	0.017		1.090	5.887 *		0.735	7.336 *
50		1.524	0.013		1.092	3.371 *		0.736	5.129 *
100		1.529	9.527 *		1.095	0.878 *		0.743	4.069 *
200		1.536	5.298 *		1.096	0.048 *		0.742	3.075 *

The symbol * indicates that the value multiply $10^{-3}$.

**Table 17:** Goodness-of-fit measures, MLEs and SEs (into parentheses) for D1.

Model	CVM	AD	KS	KS *p*-Value	MLEs and (SEs)	
HLIR	0.0513	0.3895	0.0596	0.9668	3.6538	10.2773
(α,λ)					(0.2197)	(2.5587)
TIITLIR	0.0908	0.6421	0.0776	0.7993	2.7966	10.2992
(α,θ)					(0.1574)	(2.8538)
TIR	0.1767	1.2000	0.2540	0.0002	7.5093	0.8891
(θ,λ)					(19.4108)	(0.0182)
OFIR	0.5913	3.7026	0.1801	0.0227	2.9540	1.3910
(θ,α)					(0.1862)	(0.1231)
IR	0.1875	1.2706	0.3549	0.0000	2.2827	-
(α)					(0.1374)	-

**Table 18:** Goodness-of-fit measures, MLEs and SEs (into parentheses) for D2.

Model	CVM	AD	KS	KS *p*-Value	MLEs and (SEs)	
HLIR	0.0476	0.2821	0.0626	0.9745	9.0540	1.5057
(α,λ)					(0.8159)	(0.2381)
TIITLIR	0.0487	0.2904	0.0726	0.9142	7.1702	1.2912
(α,θ)					(0.5758)	(0.2319)
TIR	0.0496	0.2885	0.0659	0.9598	105.9762	0.4105
(θ,λ)					(19.4108)	(0.3493)
OFIR	0.0964	0.7205	0.1458	0.1623	47.0580	0.7820
(θ,α)					(5.1027)	(0.0838)
IR	0.0488	0.2934	0.0821	0.8203	9.3593	-
(α)					(0.6092)	-

**Table 19:** Goodness-of-fit measures based on the log-likelihood for D1.

Distribution	$-\hat{\ell }$	AIC	CAIC	BIC	HQIC
HLIR	50.5018	105.0030	105.1856	109.4720	106.7764
TIITLIR	52.0685	108.1371	108.3189	112.6053	109.9098
TIR	71.9390	145.8787	145.9384	148.1128	146.7651
OFIR	71.7113	147.4228	147.6046	151.8910	149.1955
IR	88.4130	178.8262	178.8859	181.0603	179.7125

**Table 20:** Goodness-of-fit measures based on the log-likelihood for D2.

Distribution	$-\hat{\ell }$	AIC	CAIC	BIC	HQIC
HLIR	188.4997	380.9994	381.2137	385.1545	382.6213
TIITLIR	188.6142	381.2283	381.4426	385.3834	382.8503
TIR	189.1310	382.2620	382.4762	386.4170	383.8839
OFIR	193.7239	391.4479	391.6621	395.6029	393.0698
IR	190.5877	383.1754	382.2455	385.2529	382.9863

## References

[1] Trayer V.N. (1964). Proceedings of the Academy of Science Belarus.

[2] Voda V.G. (1972). On the inverse Rayleigh distributed random variable. Rep. Stat. Appl. Res..

[3] Gharraph M.K. (1993). Comparison of estimators of location measures of an inverse Rayleigh distribution. Egypt Stat. J..

[4] El-Helbawy A.A. (2005). Bayesian estimation and prediction for the inverse Rayleigh lifetime distribution. Proceeding of the 40st Annual Conference of Statistics, Computer Sciences and Operation Research.

[5] Mohsin M., Shahbaz M.Q. (2005). Comparison of negative moment estimator with maximum likelihood estimator of inverse Rayleigh distribution. Pak. J. Stat. Oper. Res..

[6] Soliman A., Amin E.A., Abd-EI Aziz A.A. (2010). Estimation and prediction from inverse Rayleigh distribution based on lower record values. Appl. Math. Sci..

[7] Dey S. (2012). Bayesian estimation of the parameter and reliability function of an inverse Rayleigh distribution. Malays. J. Math. Sci..

[8] Sindhu T.N., Aslam M., Feroze N. (2013). Bayes estimation of the parameters of the inverse Rayleigh distribution for left censored data. Prob. Stat. Forum.

[9] Fan G. (2015). Bayes estimation for inverse Rayleigh model under different loss functions. Res. J. Appl. Sci. Eng. Technol..

[10] Rasheed H.A., Ismail S.Z., Jabir A.G. (2015). A comparison of the classical estimators with the Bayes estimators of one parameter inverse Rayleigh distribution. Int. J. Adv. Res..

[11] Panwar M.S., Sudhir B.A., Bundel R., Tomer S.K. (2015). Parameter estimation of Inverse Rayleigh distribution under competing risk model for masked data. J. Inst. Sci. Technol..

[12] Rasheed H.A., Aref R.K.H. (2016). Reliability estimation in inverse Rayleigh distribution using precautionary loss function. Math. Stat. J..

[13] Leao J., Saulo H., Bourguignon M., Cintra J., Rego L., Cordeiro G.M. (2013). On some properties of the beta Inverse Rayleigh distribution. Chil. J. Stat..

[14] Ahmad A., Ahmad S.P., Ahmed A. (2014). Transmuted inverse Rayleigh distribution: A generalization of the inverse Rayleigh distribution. Math. Theory Model..

[15] Khan M.S. (2014). Modified inverse Rayleigh distribution. Int. J. Comput. Appl..

[16] Khan M.S., King R. (2015). Transmuted modified inverse Rayleigh distribution. Austrian J. Stat..

[17] Haq M.A. (2015). Transmuted exponentiated inverse Rayleigh distribution. J. Stat. Appl. Prob..

[18] Haq M.A. (2016). Kumaraswamy exponentiated inverse Rayleigh distribution. Math. Theory Model..

[19] Fatima K., Ahmad S.P. (2017). Weighted inverse Rayleigh distribution. Int. J. Stat. Syst..

[20] Elgarhy M., Alrajhi S. (2019). The odd Fréchet inverse Rayleigh distribution: Statistical properties and applications. J. Nonlinear Sci. Appl..

[21] Mohammed H.F., Yahia N. (2019). On type II Topp-Leone inverse Rayleigh distribution. Appl. Math. Sci..

[22] Yahia N., Mohammed H.F. (2019). The type II Topp-Leone generalized inverse Rayleigh distribution. Int. J. Contemp. Math. Sci..

[23] Rao G.S., Mbwambo S. (2019). Exponentiated inverse Rayleigh distribution and an application to coating weights of iron sheets data. J. Probab. Stat..

[24] Cordeiro G.M., Alizadeh M., Marinho E.P.R.D. (2016). The type I half-logistic family of distributions. J. Stat. Comput. Simul..

[25] Anwar M., Zahoor J. (2018). The half-logistic Lomax distribution for lifetime modeling. J. Probab. Stat..

[26] Aldahlan M.A. (2019). Different methods of estimation for the parameters of half logistic Lomax distribution. Appl. Math. Sci..

[27] Elbatal I., Almarashi A.M., Elgarhy M., Haq M.A. (2019). Type I half-logistic power Lindley distribution with applications. Far East J. Math. Sci. (FJMS).

[28] Anwar M., Bibi A. (2018). The Half-Logistic generalized Weibull distribution. J. Probab. Stat..

[29] Shrahili M., Elbatal M., Muhammad M. (2019). The type I half-logistic Burr X distribution: Theory and practice. J. Nonlinear Sci. Appl..

[30] ZeinEldin R.A., Chesneau C., Jamal F., Elgarhy M. (2019). Different estimation methods for Type I half-logistic Topp-Leone distribution. Mathematics.

[31] Klein J.P., Moeschberger M.L. (2003). Survival Analysis: Techniques for Censored and Truncated Data.

[32] Rady E.A., Hassanein W.A., Elhaddad T.A. (2016). The Power Lomax Distribution with an Application to Bladder Cancer Data.

[33] Butler R., McDonald J. (1989). Using incomplete moments to measure inequality. J. Econom..

[34] Cowell F.A. (1995). Measuring Inequality.

[35] Kenney J.F., Keeping E.S. (1962). Mathematics of Statistics.

[36] Moors J.J.A. (1988). A quantile alternative for kurtosis. J. R. Stat. Soc. Ser..

[37] Rényi A. On measures of entropy and information. Proceedings of the 4th Berkeley Symposium on Mathematical Statistics and Probability.

[38] Tsallis C. (1988). Possible generalization of Boltzmann-Gibbs statistics. J. Stat. Phys..

[39] Amigo J.M., Balogh S.G., Hernández S. (2018). A brief review of generalized entropies. Entropy.

[40] Ahsanullah M., Kibria B.M.G., Shakil M. (2014). Normal and Student’s T Distributions and Their Applications.

[41] Casella G., Berger R.L. (2002). Statistical Inference.

[42] Swain J., Venkatraman S., Wilson J. (1988). Least squares estimation of distribution function in Johnson’s translation system. J. Stat. Comput. Simul..

[43] Kao J.H. (1958). Computer methods for estimating Weibull parameters in reliability studies. IRE Trans. Reliab. Qual. Control..

[44] Kao J.H. (1959). A graphical estimation of mixed Weibull parameters in life-testing of electron tubes. Technometrics.

[45] D’Agostino R., Stephens M. (1986). Goodness-of-Fit Techniques.

[46] Luceno A. (2006). Fitting the generalized Pareto distribution to data using maximum goodness-of-fit estimators. Comput. Stat. Data Anal..

[47] Macdonald P.D.M. (1971). Comment on an estimation procedure for mixtures of distributions by Choi and Bulgren. J. R. Stat. Soc. B.

[48] Kundu D., Raqab M.Z. (2009). Estimation of R = P(X < Y) for three parameter Weibull distribution. Statist. Probab. Lett..

[49] Mead M.E. (2016). On five- parameter Lomax distribution: Properties and applications. Pak. J. Stat. Oper. Res..

[50] Claeskens G., Hjort N.L. (2008). Model Selection and Model Averaging.

[51] Marinho P.R.D., Silva R.B., Bourguignon M., Cordeiro G.M., Nadarajah S. (2019). AdequacyModel: An R package for probability distributions and general purpose optimization. PLoS ONE.

